# Uterine Fibroids: Some Facts in Regard to These Neoplasms1Read at the meeting of the Central New York Medical Association, Held in Rochester, June 16, 1893.

**Published:** 1893-09

**Authors:** Henry D. Ingraham

**Affiliations:** Professor of Diseases of Women and Children, Niagara University, Buffalo, N. Y.


### UTERINE FIBROIDS: SOME FACTS IN REGARD TO THESE NEOPLASMS.[1]

By HENRY D. INGRAHAM, M. D.,

Professor of Diseases of Women and Children, Niagara University, Buffalo, N. Y.

Although several theories have been advanced with reference to the growth and development of these neoplasms, yet our knowledge of their etiology is yet very imperfect. That they are of frequent occurrence no one will deny, one writer claiming that one-fifth of all women over thirty-five have fibroids. They occur in all classes, being fully as common in the wealthier as in the poorer class. The general belief that they are more frequent in the colored race remains to be proven. While the greater number of them may exist unnoticed, many of them are of more serious import, often causing much discomfort and suffering, and frequently leading to conditions which result in the death of the patient.

Any woman who has a uterine fibroid, even though it does not trouble her, or inconvenience her in any way at present, may, at any time, develop not only unpleasant and uncomfortable symptoms, but even dangerous ones. These growths are a perpetual source of danger.

Whatever may be the seat of the fibroid, it provokes a constant but varying degree of uterine hypertrophy, and, hence, almost numberless and varying symptoms. The location and shape of the

---

1. Read at the meeting of the Central New York Medical Association, held in Rochester, June 16, 1893.

growth frequently are greater sources of discomfort and danger than the size, owing to its interference with the functions of the adjacent organs. Often a small tumor produces much more disturbance than a larger one. The changes and alterations that may take place in the tumor itself are numerous, but I shall mention only a few of them.

Formerly, it was thought that at the menopause these growths diminished in volume and underwent a senile involution and atrophy. No doubt, this change occurs in the greater portion of these neoplasms, yet others suddenly begin to grow or develop changes that result in the death of the patient, unless relieved by appropriate means. Sometimes before the menopause, without any apparent reason, or the result of any treatment, these growths undergo involution, shrink, and completely disappear, except a cicatricial tissue, and cause no further disturbance. Softening may result from various causes, the most frequent one being pregnancy, due to the exaggerated nutrition of the uterus, which occurs at this time. Fatty degeneration also occurs, although rarely, and most frequently after pregnancy. Calcification, although an unusual change, does occur. It is now a well-established fact that malignant degeneration of these growths occurs, although formerly it was denied that this change ever took place.

It is not, however, so much the changes that take place in the fibroids themselves as the changes that occur in the adjacent organs as the result of the pressure of these growths. And here let me again state that the small tumor is just as likely to cause complications as the large one. If the tumor be small and nodular, the complications come on early, due to the irregular pressure upon the surrounding organs ; but if it be symmetrical, the complications do not appear until the tumor has attained much greater dimensions.

The former class seems to be much more sensitive and intolerant of interference than the latter, and any form of palliative treatment may, at any time, light up symptoms not only urgent but dangerous. The pain, due to pressure upon the bladder or other surrounding organs, may be sufficient to render the life of the poor sufferer unbearable, even though there be no organic change in the structure of these organs. Pressure and irritation often cause peritonitis, and, although it does not usually terminate fatally, it renders the patient an invalid for life, and seriously complicates any operative procedure. Tubal disease, in some one of its various forms, is a frequent complication of fibroids, and this condition should be borne in mind when treatment is considered. Organic disease of the heart is quite frequently a complication of these growths, and although this is an indication for operative interference, yet the fact of its existence tends to render the operation more dangerous.

The most important question is, What shall be done for the relief of this class of patients? The use of ergot, to cause the shrinkage of the growths, which was so popular a few years ago, is rarely resorted to at present. The use of galvanism, which was prominently brought to the notice of the profession by Apostoli, and which was thoroughly tried by many faithful followers, has been abandoned by nearly every one. At one time I used it quite extensively; in some cases causing a shrinkage of the tumor; in others, relieving many of the unpleasant symptoms, especially the hemorrhage. Yet it is doubtful if the patients were relieved more than they would have been by other means, such as tonics, ergot, and the use of the curette. I had the opportunity, three years ago, of observing its effects as used by Apostoli himself, and the most that he did, or claimed for it, was the relief of the symptoms, and this was done only in a portion of the cases, as any one can readily appreciate when the complications are understood.

Operative measures for the relief of fibroids have not generally been resorted to, because of the great mortality resulting from the attempt to remove the tumors ; and the results obtained by the removal of the tubes and ovaries have not been as satisfactory as could be wished. Yet, for the past two years, the mortality with several operators has been small, and, no doubt, the time is not far distant when a uterine fibroid can be removed as safely, by a competent operator, as an ovarian cyst. I am well aware that it is a much more difficult operation, but, with our improved technique, the removal is perfectly justifiable and the results satisfactory. This may seem an exaggerated statement to many ; it certainly will to that class of physicians who advise patients that it is unnecessary and too dangerous to have tubes removed that are an inch or more in diameter and, probably, filled with pus.

At present, several methods for the removal of fibroids are being used, but the ones that are attracting the most attention are total extirpation, as practised and chiefly brought to the notice of the profession at large by Dr. Martin, of Berlin, and, in this country, by Dr. Polk, of New York, and the method of ligating the ovarian and uterine arteries, and amputating the cervix and leaving a small part of it. The latter operation was first brought to the notice of the profession by Dr. Baer, of Philadelphia, and is known in this country as his operation. It is claimed for this method that it is easier to perform than total extirpation, and that it leaves the vagina in better condition, as it does not take away any portion of it, and the support is much stronger than when total extirpation is done. Either one of these methods gives much better results, at the present time, than any other manner of operating. Which one, if either, will finally be generally adopted by operators, trial alone will determine. At the present time I incline to the Baer method, although my results with it have not been as good as with total extirpation. I have done only two operations by this method, and both my patients died; but I do not think it the fault of the method. Dr. Baer has done this operation thirty times, with only two deaths, both the result of other causes, and not due to the method of operating. Prof. Winckel, of Munich, is reported to have done this operation fifty-one times, with only one death. The method of total extirpation, in the hands of Drs. Martin and Polk, and some others, has given about the same results as those obtained by Dr. Baer. Enough has been done to demonstrate that, in the hands of competent operators, the removal of uterine fibroids, that render the patient an invalid, is perfectly justifiable, and that the mortality should not be above six or seven per cent. Some put it even lower.

